# Cardiovascular Involvement in Kawasaki Disease Is Much More Than Mere Coronary Arteritis

**DOI:** 10.3389/fped.2020.526969

**Published:** 2020-09-24

**Authors:** Rakesh Kumar Pilania, Ankur Kumar Jindal, Dharmagat Bhattarai, Sanjeev Hanumantacharya Naganur, Surjit Singh

**Affiliations:** ^1^Allergy Immunology Unit, Department of Paediatrics, Advances Paediatrics Centre, Post Graduate Institute of Medical Education and Research, Chandigarh, India; ^2^Department of Cardiology, Advances Cardiac Centre, Post Graduate Institute of Medical Education and Research, Chandigarh, India

**Keywords:** cardiac biomarkers, echocardiography, Kawasaki disease, Kawasaki disease shock syndrome, myocarditis, pericarditis

## Abstract

Kawasaki disease (KD) is now a common cause of acquired heart disease in children. Coronary artery involvement is the most serious complication in children with KD. Several non-coronary complications have now been identified in this condition but these are often overlooked. Myocarditis is an integral component of KD and may be more common than coronary artery abnormalities. Pericardial involvement and valvular abnormalities have also been observed in patients with KD. KD shock syndrome is now being increasingly recognized and may be difficult to differentiate clinically from toxic shock syndrome. Endothelial dysfunction has been reported both during acute stage and also on follow-up. This may be a potentially modifiable cardiovascular risk factor.

## Introduction

Kawasaki disease (KD) is one of the commonest vasculitides in children (1, 2). At time of its first recognition in 1967 by Dr. Tomisaku Kawasaki, it was described as “*mucocutaneous lymph node syndrome*” and was considered as a benign disease with self-limiting course (3). However, autopsy studies later revealed the coronary artery complications associated with KD (4). Over time, it has now been realized that KD may cause several other cardiac complications as well (5, 6) (Table 1). It has been shown that myocarditis in KD is, in fact, more common than coronary artery involvement and may be almost universal (7). In this review, we have discussed various non-coronary cardiac complications in patients with KD.

**Table 1:** Cardiovascular complications of Kawasaki disease (KD).

**S. no**	**Complication**
1.	Coronary artery abnormalities (dilatation, aneurysm)
2.	Coronary artery aneurysms and thrombosis
3.	Coronary stenosis
4.	Coronary calcification
5.	Myocarditis
6.	Myocardial infarction
7.	Kawasaki disease shock syndrome
8.	Pericarditis, pericardial effusion and cardiac tamponade
9.	Cardiac fibrosis
10.	Cardiomyopathy
11.	Endothelial dysfunction
12.	Systemic artery aneurysms

## Myocarditis

Myocarditis appears to be an integral part of KD and may be seen in all patients (7, 8). Fujiwara et al. in 1978 have reported autopsy studies on 20 patients with KD (4). The authors classified pathology of KD into four clinico-pathological stages and noted pancarditis on histology. In the first 9 days of illness, predominant finding was carditis associated with edema and inflammatory cell infiltrate in all three layers of heart (4). Yutani et al. have performed right ventricular biopsy in 201 patients with KD after periods ranging from 1 month to 11 years of diagnosis of KD (9). They showed that myocarditis and fibrotic changes were seen in all patients. Sequelae of myocarditis were evident even during follow-up (9). Similarly, Yonesaka et al. have performed subendocardial myocardial biopsies and showed that findings of myocyte disarray, interstitial fibrosis and myocardial cell degeneration persist in patients with KD on follow-up (10). Necropsies have shown that patients with KD developed diffuse myocardial fibrosis and increased expression of transforming growth factor (TGF)-β in wall of coronary artery aneurysm (11). These studies help explain the pathogenesis of myocardial fibrosis/cardiomyopathy in children with KD on follow-up (9–12).

In 1995, Anderson et al. had published the long term effects of KD on cardiac function in 67 patients. Authors performed serial M-mode echocardiograms at baseline, 1–3, 3–12 months, and after 1 year of diagnosis. This study showed that left atrial and left ventricular dimensions continued to be abnormal in more than 50% of patients even 1 year after KD. Fractional shortening was abnormal initially but normalized at 3 months of follow-up. Left ventricular emptying was significantly reduced. Moreover, almost a third of patients evaluated beyond 1 year had diastolic dysfunction. This was amongst the first few studies showing that patients with KD had abnormalities in cardiac functions even in absence of CAAs (13).

Nakaoka et al. have recently reported on cardiac function in patients with KD having asymptomatic coronary artery disease by cardiac magnetic resonance (CMR) imaging. It was found that transmural extent of late gadolinium enhancement in this subgroup was ≤50% and these patients had subendocardial infarction with normal left ventricular function (14).

### Pathophysiology

KD myocarditis often develops as a result of acute or subacute inflammation of interstitial tissue of myocardium and is usually concentrated around the coronaries. Myocardial inflammation peaks by day 10 of illness and gradually subsides by end of 3 weeks. During this stage, there is inflammation of small arteries of the myocardium including perivasculitis. Inflammation in interstitial tissue of the myocardium develops as a result of spill of inflammatory cells from perivasculitis. This explains prompt recovery of myocardial function following administration of intravenous immunoglobulin (IVIg) in these patients. Pathophysiology of KD myocarditis, therefore, differs in several aspects from viral myocarditis. While viral myocarditis is characterized by predominant lymphocytic interstitial cell infiltrates, edema and myocyte or myocardial fiber bundle necrosis, KD myocarditis on other hands is characterized by myocardial interstitial edema, vasodilatation and inflammatory cell infiltration. Severe myocarditis in patents with KD can manifest independent of coronary artery involvement. Rarely, patents with KD and severe inflammatory myocardial inflammation can have degenerative changes resulting in cardiomyopathy (8, 15, 16).

### Clinical Characteristics

Myocarditis, which is one of the earliest presentations of KD, usually presents within first 10 days of illness in contrast to coronary artery vasculitis and coronary artery abnormalities (CAAs) that usually develop after day 10 of illness (8, 15). Audible gallop, tachycardia and hyperdynamic precordium are the subtle clinical correlates of KD myocarditis. Strain abnormalities and evidence of systolic and diastolic dysfunction are correlates of KD myocarditis on echocardiography (8).

### Asymptomatic Myocarditis

Myocarditis in patients with KD is often asymptomatic and can easily be missed (6, 8).

### Symptomatic Myocarditis

Myocarditis in KD can present clinically as unexplained tachycardia, congestive cardiac failure, hemodynamic instability, requirement of inotropic support and arrhythmias (17–23). Symptomatic myocarditis remains a significant cause of morbidity and mortality during the initial phase of the KD (15, 24–26).

### Viral Myocarditis vs. KD Associated Myocarditis

Myocarditis of KD needs to be differentiated from viral myocarditis:

- Viral myocarditis generally follows a prodrome and at time of clinical presentation patients are usually afebrile. Myocarditis in KD develops in the early phase of disease and is usually accompanied by high grade fever (8).
- Myocardial dysfunction in KD is usually transient and responds dramatically to anti-inflammatory therapy with intravenous immunoglobulin (IVIg) (8, 15). Response to IVIg in viral myocarditis is, at best, modest (27, 28).
- The pathological changes in KD largely consist of interstitial edema and inflammatory cell infiltrate, while in viral myocarditis cell necrosis is the predominant finding (15).

### Biomarkers for Myocarditis

Several biomarkers have been proposed in patients with KD to evaluate myocardial dysfunction and injury. The biomarker that has shown clinical promise is N-terminal pro B-type natriuretic peptide (NT-proBNP) (8, 29, 30).

#### NT-proBNP

In response to volume and pressure cardiac overload, pre-pro-BNP is synthesized and processed to pro-BNP. Pro-BNP is then processed to biologically active BNP fragment, and NT-pro-BNP which is inert. NT-ProBNP is preferred to BNP as a biomarker for laboratory assays as it has a longer half-life. Synthesis of pro-BNP from cardiac myocytes is controlled by many factors including mechanical factors like dilatation and strain of cardiac chambers, various neurohormonal factors and cytokines (e.g., interleukin-1 β or tumor necrosis factor α). Interpretation of pro-BNP levels is difficult as it can be affected by several factors other than myocardial damage. Pro-BNP levels are age dependent and are highest in infancy and early childhood (31). Presence of acute kidney injury and decreased glomerular filtration is also associated with falsely elevated pro-BNP levels (30). Several studies have found NT-ProBNP to be a useful marker for diagnosis as well as for assessment of disease severity in KD (29, 32, 33). Age specific cut-off values have been calculated and Z scores are also available for assessment of elevated levels of pro-BNP (29, 31). Reddy et al. have assayed levels of pro-BNP during the acute stage of KD. The authors reported that levels above 1025 pg/ml have a specificity of 96% and sensitivity of 88% for diagnosis of KD (33).

Studies have shown a positive correlation of NT-pro-BNP with C-reactive protein and hypoalbuminemia in children with KD during initial phase of disease. NT-pro-BNP levels are significantly raised during the acute phase of KD when compared to controls (32, 34). Levels of NT-pro-BNP showed negative correlation with left ventricular (LV) ejection fraction, fractional shortening, cardiac index values, diastolic function and positive correlation with impairment in ventricular relaxation (32, 34).

NT-pro-BNP is a valuable assessment tool in clinical evaluation of patients with incomplete forms of KD (29, 35, 36). Dionne et al. have proposed a diagnostic algorithm based on NT-pro-BNP. This is very useful in patients with incomplete forms of KD (29).

#### Cardiac Troponins

Serum cardiac troponins are superior to creatine kinase (CK)-MB for detection of myocardial damage in myocarditis (37). Kim et al. have compared cardiac troponin I and CK-MB levels in 45 patients with KD. Authors showed that levels of cardiac troponin I were elevated in 18 (40%) patients, while CK-MB levels were elevated in 11 (24%) patients (38).

Sato et al. have measured cardiac troponin by a highly sensitive assay and shown that cardiac troponin levels are elevated in 1/3rd of children with KD during the acute phase. These levels may continue to remain elevated during the convalescent phase as well (34). However, levels of cardiac troponin have very weak correlation with NT-pro-BNP and there was no significant correlation with systolic or diastolic function or CAAs in patients with KD (34). Checchia et al. have shown that elevation of cardiac troponin I in patients with KD was not significant and there was no significant correlation with development of CAAs (39).

#### Other Cardiac Biomarkers

Soluble suppression of tumorigenicity-2 (ST2) belongs to the IL-1 receptor family. It is released by cardiomyocytes and fibroblasts during stress phase of KD myocarditis. It has been reported to be positively correlated with impairment of ventricular relaxation in patients with KD (27). Gamma-glutamyl transferase and alanine transferase, however, have not been found to be useful in establishing a clinical diagnosis of KD (40).

### Imaging in Myocarditis

#### Echocardiographic Features

**Figure 1:**
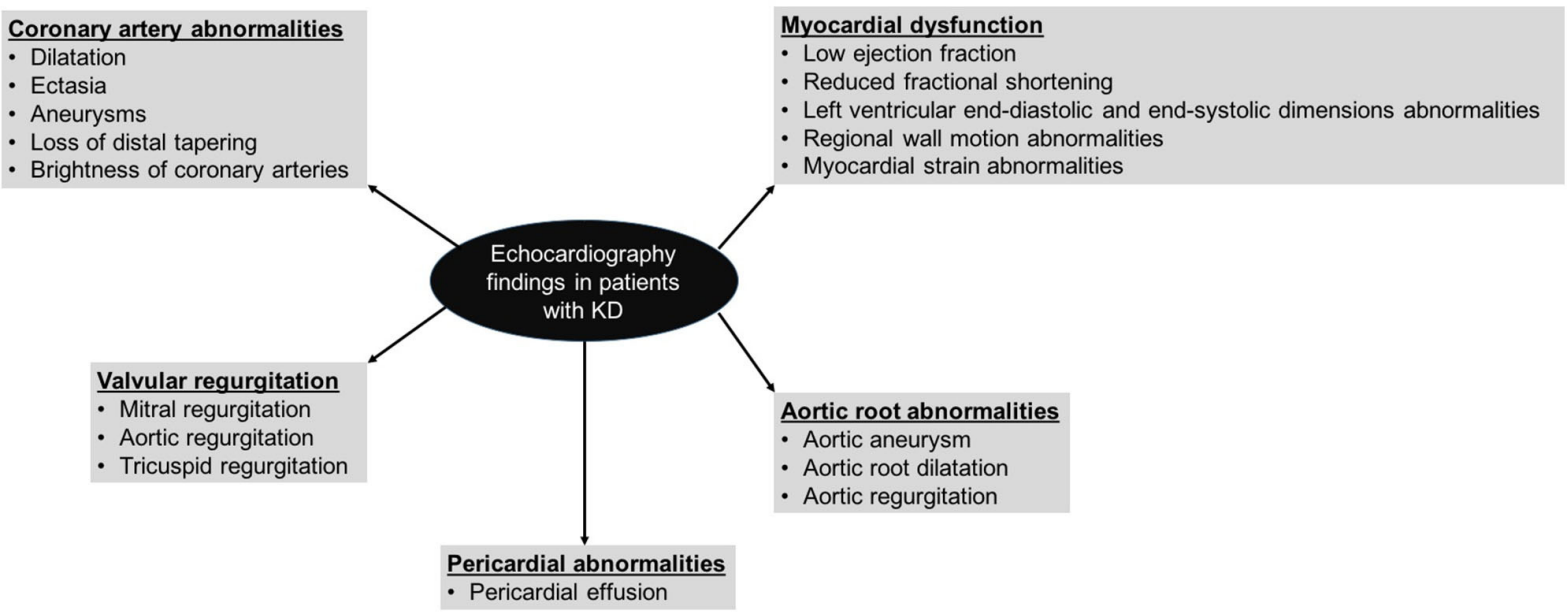
Echocardiography findings in patients with Kawasaki disease.

2D-echocardiography remains the mainstay of imaging for cardiovascular assessment in KD both during acute phase and long-term follow-up (1, 2, 36, 41, 42). It cannot be overemphasized that cardiac assessment in patients with KD is just not limited to coronary artery assessment and detailed cardiac assessment for ventricular functions, wall motion abnormalities, valvular functions, and pericardial effusion need to be performed as well (Figure 1). Traditional echocardiographic evaluation of KD myocarditis by M mode includes parameters for left ventricular systolic dysfunction and left ventricular dilatation. The diastolic function of the heart is assessed by inflow parameters across both atrio-ventricular valves by pulse wave doppler and tissue doppler.

Myocarditis is universal in almost all patients with KD during the acute phase of disease. Transient left ventricular dysfunction can occur in more than 50% patients (43). Newburger et al. showed that left ventricular dysfunction in patients with KD appears unrelated to development of CAAs (44). Normal systolic function is restored after recovery from acute illness in most of the patients with KD. However, diastolic dysfunction has also been found in many studies. Therefore, ventricular function assessment should be an integral part of echocardiographic evaluation in children with KD. This should include assessment of regional wall motion abnormalities that are often a surrogate marker for coronary artery involvement (1, 41).

Speckle tracking echocardiography (STE) is a sensitive tool that can accurately detect myocardial strain and can quantify myocardial function with high reproducibility. More sensitive measures of myocardial deformation, such as *global longitudinal strain, circumferential strain*, and *strain rate* have been reported to be decreased in KD (45). Strain abnormalities in patients with KD have been seen even in absence of apparent systolic function abnormalities.

Xu et al. have shown that left ventricular systolic strain decreased significantly in children with KD during acute phase of disease. However, it improved rapidly after IVIg therapy and normalized by 6–8 weeks (46). Regional left ventricular strain was found to be impaired in basal infero-septal, basal anterolateral, apical septal, and apical inferior segments in patients with KD during midterm follow up when compared with controls (47). Left atrial strain is a well-recognized surrogate marker for raised left ventricular end diastolic pressure and left ventricular diastolic dysfunction. Lower values of left atrial strain have been reported during the acute stage in KD and this may improve during follow-up (48). Studies have reported correlation between depressed strain and disease severity (49, 50). Strain imaging may also be useful during follow-up of these patients (51). Newburger et al. showed that velocity of circumferential fiber shortening corrected for wall stress, was reduced in patients with KD during and up to 3 months after acute illness and improved spontaneously by 1 year (44).

Dedeoglu et al. have evaluated myocardial deformation at 6 months follow-up and measured global as well as regional myocardial strain by STE and showed impaired left ventricular strain in patients with KD in basal and apical segments. However, there was no association between LV dysfunction and CAAs (47). Wang et al. compared STE findings in patients with IVIg resistant KD and IVIg responsive patients with KD. It was found that the former had more severe ventricular dysfunction (52).

To conclude, mere assessment of “Z” scores of coronary arteries in children with KD is not enough. Attempts should be made to look for abnormalities of myocardial function. It is also apparent that myocardial dysfunction in KD can occur independent of coronary artery involvement.

#### Other Imaging Modalities

There are several inherent limitations associated with 2D-echocardiography. It is highly observer dependent and the results are not always reproducible (36, 41). Studies using nuclear scans have shown that myocardial inflammation can be seen in more than 50% of patients. We have published our experience on exercise myocardial perfusion scintigraphy on 84 patients with KD at least 1 month after onset of illness (53). In this study, 12 (14.3%) patients showed reversible perfusion defects and these can be seen even in patients with no demonstrable CAAs on echocardiography (53).

Tacke et al. have evaluated cardiac function in children with KD at follow-up and showed that there was no significant difference in cardiac function and fibrosis in patients with KD compared to controls while using CMR at long-term follow-up (54). In a more recent study, Bratis et al. have reported LV myocardial deformation indices using CMR and found that there was reduced myocardial strain values during the convalescent phase and this was irrespective of coronary artery involvement (55). Clearly, we need more studies to fully comprehend the residual effects of KD on the myocardium.

### ECG Features

Several conduction and repolarization abnormalities have been reported in patients with KD. These include non-specific ST and T-wave changes, PR interval prolongation, QT dispersion abnormalities and arrhythmias. In presence of severe myocarditis/pericarditis, low voltage complexes, and symptomatic arrhythmias may be seen (56–58). Bifid T-wave in limb leads have also been noted during the acute phase of KD (59). QT dispersion abnormalities may persist for several months (56, 57). Persistence of repolarization abnormalities in follow-up may indicate higher risk of ventricular arrhythmia during follow-up of patients with KD even in absence of obvious echocardiographic abnormalities (60).

### Long Term Complications of Myocarditis

In conclusion, while it is likely that most children with KD myocarditis would remain well on follow-up and attain normal systolic function, a few patients may go on to develop myocardial dysfunction, fibrosis, myocardial infarction later in life. Further, these manifestations may occur even in patients who have had no obvious CAAs (7, 8, 11).

## KD Shock Syndrome (KDSS)

KDSS is said to be occur when a patient with clinical diagnosis of KD develops systolic hypotension or shock. Although shock during acute stage of KD was recognized more than two decades ago (61–63), Kanegaye et al. defined KDSS for the first time in 2009 (64). In this study, hemodynamic instability was observed in 13/187 (7%) patients with KD (64). Since then, this entity has been reported from several centers across the world (65–67). These patients are often misdiagnosed as toxic shock syndrome (TSS) and this led to delays in institution of appropriate therapy (66, 67). KDSS is usually seen in older children and is more commonly reported in boys (66, 68, 69), although some studies have also noted a female predominance (70). Some authorities believe that KDSS may, in fact, be a unique subtype of KD (65). KDSS still remains an under-recognized complication (71).

The etiopathogenesis of KDSS remains poorly understood. It involves a combination of myocardial dysfunction (secondary to myocarditis) and distributive shock (caused by increased vascular permeability which is secondary to dysregulated cytokine storm) (8, 66, 72).

Patients with KDSS have been reported to have increased incidence of gastrointestinal manifestations, hyponatremia, anemia, thrombocytopenia, hypoalbuminemia, elevated inflammatory markers (e.g., neutrophila, high CRP, ESR), IVIg resistance, CAAs (up to 65%), morbidity and mortality (up to 6.8%) (65, 68, 69, 73). In addition, levels of several cytokines (e.g., TNF-α, interferon-γ) are found to be elevated in patients with KDSS (74). Whether these biomarkers can be considered for early diagnosis of KDSS remains conjectural.

### How Does One Differentiate Between TSS and KDSS?

The clinical presentation of KDSS and TSS may appear similar and it may be very difficult to differentiate the two conditions at bedside. Lin et al. retrospectively analyzed 16 patients with KDSS and 17 patients with TSS (69). It was found that patients with KDSS were usually younger and had less prominent gastrointestinal symptoms. While anemia and thrombocytosis were more commonly seen in patients with KD, lymphopenia was characteristic of TSS. Further, CAAs and valvular abnormalities were seen only in KDSS (69, 75).

### Hyper-Inflammatory Syndrome Associated With COVID-19—A Novel Syndrome

Severe acute respiratory syndrome coronavirus 2 (SARS-CoV-2) infection has rapidly spread worldwide since it was first identified in Wuhan, China in November 2019. Initial reports suggested that SARS-CoV-2 causes milder disease in children. However, by late April 2019a hyper-inflammatory syndrome had been identified (76). This was characterized by high grade persistent fever and multisystemic clinical manifestations suggesting a delayed hyperimmune response to SARS-CoV-2 infection. This novel syndrome has been variably termed as “multisystem inflammatory disorder in children and adolescents,” “multisystem inflammatory syndrome in children (MIS-C),” “pediatric inflammatory multisystem syndrome temporally associated with SARS-CoV-2 (PIMS-TS)” (77). Clinical features of this syndrome include cardiovascular collapse (e.g., hypotension, myocarditis, and myocardial dysfunction), predominant gastrointestinal symptoms (e.g., diarrhea, vomiting, and pain abdomen), features similar to KD (e.g., rash, conjunctival injection, and extremity changes), and neurological manifestations (e.g., headache, irritability, and encephalopathy). Reported data suggest that patients with MIS-C are usually older, have predominant gastrointestinal manifestations (some may present with acute surgical abdomen) and have myocardial dysfunction. Clinical findings in this syndrome may mimic those of KDSS and TSS (77–79). MIS-C and KD are probably two distinct entities as they have differences in demographic, laboratory and clinical findings (80, 81). Whittaker et al. have compared patients with PIMS-TS with KD, KDSS and TSS (82). Authors reported that patients with PIMS-TS are older than the ones in latter three categories. Further, patients with PIMS-TS were found to have higher inflammatory markers, more pronounced lymphopenia, and higher levels of troponins and NT-pro-BNP (82).

## Pericarditis

Pericarditis is a common but an under-reported manifestation in patients with KD. Gowin et al. (83) reported pericarditis in 6/30 (20%) patients while Hamza et al. (84) found pericardial involvement in 7.8% of patients with KD.

Pericardial involvement is usually mild and not clinically significant. Septate pericarditis (85) and tamponade (86–88) that may occasionally be fatal has also been reported. Cardiac tamponade may be a component of polyserositis syndrome in patients with KD or may follow rupture of one of the coronary artery aneurysms in the pericardial cavity (89). While polyserositis leading to tamponade would manifest during the acute stage, tamponade caused by rupture of aneurysm may appear at any time (90, 91) and may even manifest several years after acute KD (92). Printz et al. reported transient pericardial effusion in 3% patients of KD and it resolved by 5 weeks (93).

Mild pericarditis may resolve with IVIg and aspirin. Patients with more severe forms of pericarditis may require additional immunomodulatory therapy (94). Cardiac tamponade from coronary artery aneurysmal rupture may require urgent pericardial window and emergency coronary artery bypass grafting (89, 90).

## Valvular Abnormalities

Valvular regurgitation in acute phase has been ascribed to pancarditis, while patients having persistent valvular abnormalities are likely to have valvular dysfunction and papillary muscle dysfunction due to coronary ischemia (95). Myocardial inflammation can lead to valvular regurgitation. The most common abnormality is mitral regurgitation (MR) during acute phase of KD—this usually resolves on follow-up. It is seen commonly in patients with KD who have wall motion abnormalities or reduced ejection fraction (1, 8, 41, 93, 96). Some patients can go on to develop severe MR due to rupture of chorda tendinae. This complication may result in rapid clinical deterioration and even fatalities if not recognized and treated in time (97–99) Cardiac auscultation is, therefore, very important in patients with KD especially during the convalescent phase. Aortic regurgitation (AR) has also been reported (100, 101).

de La Harpe et al. have recently published 30 years of experience in KD and showed that 20% of patients in their cohort had valvular involvement and of these 88% had mitral valve dysfunction (96). Printz et al. have prospectively performed 2D-echocardiogtaphy on 198 patients with KD at baseline, 1 and 5 weeks of onset of illness (93). They showed that 27% of patients with KD had mild mitral regurgitation at baseline echocardiography during acute phase. Although MR had resolved significantly on follow-up at 5 weeks, 9% patients continued to have residual valvular dysfunction (93). It has, therefore, been suggested that Doppler evaluation should be a part of echocardiography evaluation in children with KD for assessment of valvular regurgitation abnormalities.

## Aortic Root Involvement

KD is a systemic vasculitis and affects several non-coronary arteries in the body. Aortic root dilatation during acute stage of KD has been reported in up to 10% patients with KD (93). Ravekes et al. assessed aortic root abnormalities in patients with KD (102). Aortic root diameters were assessed during mid-systole and at four different time points (i.e., within first 10 days, at 2, 6 weeks, and 1 year). It was observed that aortic root diameters were significantly higher as compared to controls. A significant increase in aortic root diameter was noted at 2 weeks of follow-up. Subsequently, no significant increase in aortic root diameter was noted at 6 weeks and at 1 year follow-up but the aortic root continued to remain dilated (102). Printz et al. reported aortic root dilatation at baseline, at 1 week and at 5 weeks in patients with KD (93). These authors did not report any significant change in aortic root diameters when assessed at different time intervals. Size of aortic root was found to correlate with coronary artery diameters but not with inflammatory parameters. Both studies used body surface area adjusted Z scores for assessment of aortic root diameter. AR was reported in 1–4% patients and was more common at 1 year of follow-up. Patients with AR may require valve replacement later in life (62, 103).

Increase in stiffness of aorta and decrease in elasticity has also been reported by several authors leading to functional impairment of aorta. This has been observed both during the acute stage and several years after the diagnosis of KD (104–106).

## Systemic Artery Involvement

Kato et al. first time performed angiography studies in patients with KD and revealed that 13/594 (2.2%) patients had systemic artery aneurysm (SAAs) in addition to CAAs (107). It was reported that SAAs were present only in patients who had multiple giant CAAs. Since than there have been reports of SAAs involving large arteries (e.g., iliac, femoral, subclavian, axillary). Recently Zhao et al. have reported full-body magnetic resonance angiography (MRA) or peripheral angiography in patients with KD for identification of SAAs (108). MRA (*n* = 110) was performed in patients with KD who had presumed risk factors for SAAs (e.g., patients having giant coronary aneurysm, increasing size aneurysm during acute phase or IVIg resistant KD). Peripheral angiography (*n* = 52) was performed along with CT coronary angiography in patients with giant or medium sized coronary aneurysms. Authors reported that 23/162 (14.2%) patients with KD having CAAs had SAAs, while overall prevalence was 23/1148 (2%). Most commonly affected arteries were axillary and common iliac. Risk factors for development of SAAs were young age and pronged fever (108). It appears that KD may also have a component of systemic vasculitis but this needs more detailed evaluation (107–109).

## Endothelial Dysfunction in Patients With KD

Endothelial dysfunction in KD often goes unrecognized. It is attributed to release of pro-inflammatory mediators that lead to production of reactive oxygen species. Brachial artery flow mediated dilatation (FMD) evaluation is a reliable marker for endothelial dysfunction. FMD depicts the capacity of brachial artery to increase its diameter in response to increase in blood flow. Studies have shown endothelial dysfunction of brachial artery in patients with KD that may persist even a decade after the acute stage (105, 110–115). Dietz et al. has observed the increased stiffness index patients with KD and CAAs (116). These abnormalities have also been reported in patients who have no obvious CAAs detected during acute stage of KD (117). Pulse wave velocity (PWV) is also a simple and non-invasive tool for assessment of arterial stiffness. Studies have shown that PWV is higher in patients with history of KD as compared to controls (118). Carotid intima-media thickness (cIMT), well-recognized as a surrogate marker for atherosclerosis, has been found to be significantly higher in children with KD on follow-up. These studies emphasize the need for long-term follow-up of children with KD even in situations wherein there have been no CAAs (116, 119–122).

It is conjectural whether endothelial dysfunction correlates with occurrence of CAAs (123–126). Patients with KD and CAAs have been reported to have an increased risk of myocardial infarction and early deaths (11, 116, 127–130). Recent literature has, however, suggested that long term cardiovascular morbidity in patients with KD may not only be restricted to patients who have been detected to have CAAs (11, 131). Autopsy studies have demonstrated luminal myofibroplastic proliferation in both aneurysmal as well as non-aneurysmal coronary arteries (16). Therefore, it is prudent to counsel patients with KD with regard to modifiable cardiovascular risk factors.

To conclude, it is clear that KD is associated with several cardiovascular sequelae. While CAAs are the most well-recognized complications of this condition, other affectations like myocarditis, KDSS, valvular abnormalities, and endothelial dysfunction are now being increasingly recognized. Early identification and appropriate treatment of these complications is of paramount importance.

## Author Contributions

RP and AJ: writing of initial draft of manuscript, editing and revision of manuscript at all stages of its production, and review of literature. DB and SN: contributed to editing of manuscript and review of literature. SS: inception of idea, critically revision of the manuscript at all stages of its production, final approval of manuscript, and review of literature. All authors contributed to the article and approved the submitted version.

## Conflict of Interest

The authors declare that the research was conducted in the absence of any commercial or financial relationships that could be construed as a potential conflict of interest.
